# Concurrent Presentation of an Anomalous Right Coronary Artery and an Unusual Bovine Arch: Case Report

**DOI:** 10.1016/j.jscai.2025.103759

**Published:** 2025-07-23

**Authors:** Vasiliki Tasouli-Drakou, Jasmine Dugal, Divyansh Sharma, KaChon Lei, Chowdhury Ahsan

**Affiliations:** a Department of Internal Medicine, University of Nevada, Las Vegas, Nevada; b Department of Cardiovascular Medicine, University of Nevada, Las Vegas, Nevada

**Keywords:** anomalous coronary arteries, aortic surgery, atherosclerosis, cardiovascular health, case report

## Abstract

Anomalous coronary arteries from the opposite sinus of Valsalva are rare but can increase the risk for life-threatening complications. We present the case of a 59-year-old man who was found to have an anomalous right coronary artery and a rare bovine arch variant with a single common brachiocephalic trunk, along with severe proximal left anterior descending artery stenosis on left heart catheterization. The patient underwent a minimally invasive coronary artery bypass graft and unroofing of the anomalous coronary artery. To our knowledge, this is the first reported case of these concurrent anomalies in an individual without tetralogy of Fallot.

## Background

The phenomenon of anomalous coronary arteries can be observed in coronary artery origination, overall course, or termination. When coronary arteries arise from the opposite sinus of Valsalva (ACAOS), they are classified as either L-ACAOS or R-ACAOS, depending on whether the left or right coronary artery (RCA) is involved, respectively. ACAOS cases are mostly asymptomatic but can predispose patients to symptoms and increase the risk for sudden cardiac death (SCD), especially in young adults and athletes, where 30% of SCDs are attributed to ACAOS.^1^

Anomalies in the aortic arch (AA), including bovine arches, have also been described in the literature. In type 1 and type 2 bovine arches, respectively, the left common carotid artery shares a common origin with or arises directly from the brachiocephalic trunk.^2^^,^^3^ Concurrence of anomalous coronary arteries and anomalous variants, including bovine arches, has not been described in the literature. Nonetheless, individuals with tetralogy of Fallot are predisposed to coronary artery and AA anomalies; hence, their concurrence cannot be excluded.^4^

## Case presentation

**Figure 1:**
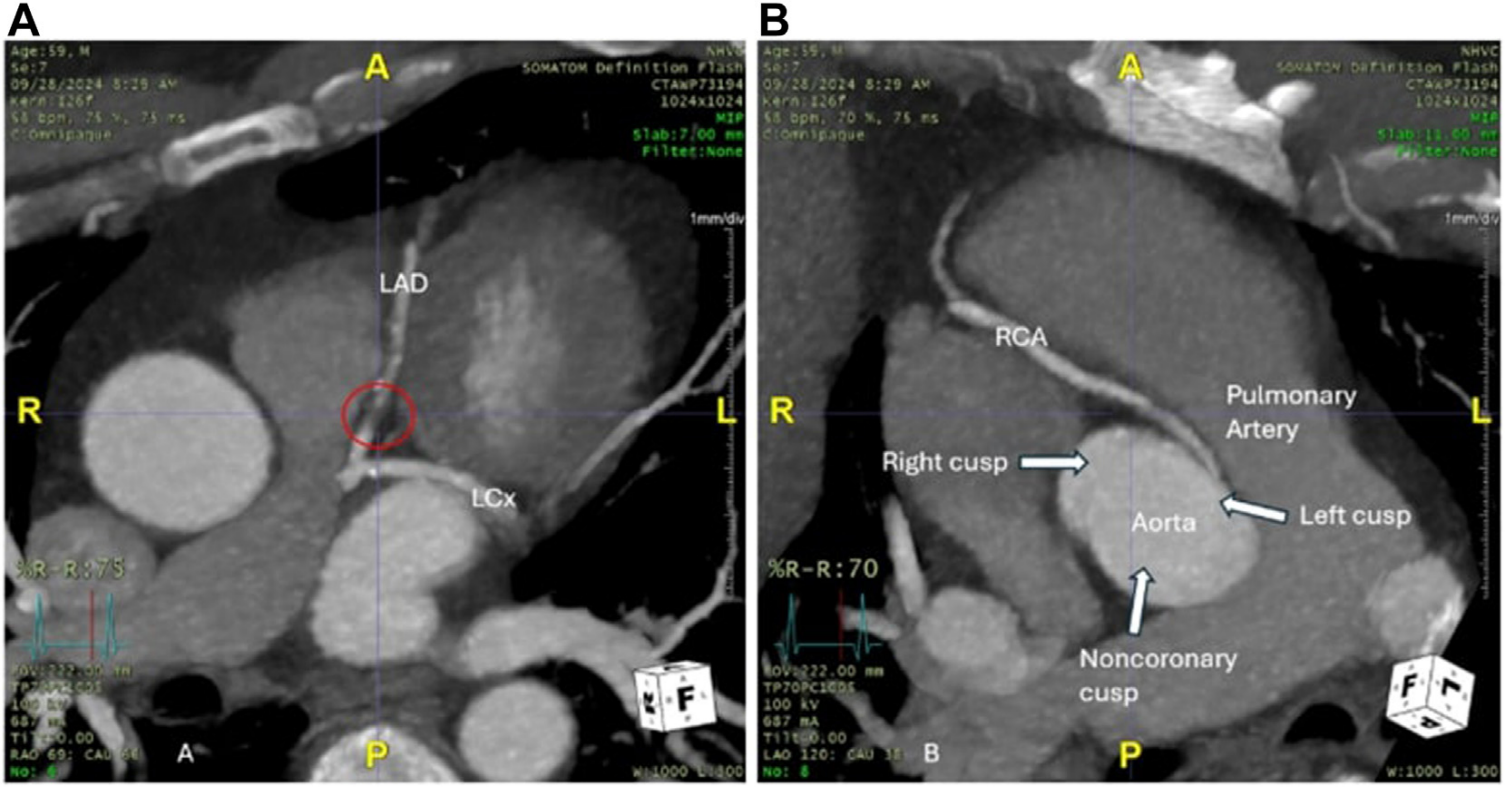
**Outpatient coronary computed tomography angiography images.** (A) The soft, microcalcified plaque in the left anterior descending coronary artery (LAD) is circled. (B) An anomalous right coronary artery (RCA) can be seen arising from the left cusp. Its course is intramural (attached to the cardiac muscle) and interarterial (between the aorta and the pulmonary artery). LCx, left circumflex artery.

Our case involves a 59-year-old Hispanic man with a medical history of hypertension, diabetes, and hyperlipidemia who presented for an elective left heart catheterization (LHC) for chest pain on exertion for multiple years. The social history was unremarkable. An outpatient regadenoson stress test suggested normal wall motion, a possible anterior artifact, and a minimal amount of ischemia of the septal and apical walls; however, it was nondiagnostic overall. A follow-up coronary computed tomography angiography showed a small, calcified plaque in the left circumflex artery (LCx), a microcalcified plaque in the left anterior descending (LAD) ( Figure 1A), and an anomalous RCA with a malignant intramural and interarterial (between the aorta and the pulmonary artery) course (Figure 1B). No other outpatient imaging, such as a cardiac magnetic resonance imaging, was performed. Instead, a LHC was recommended to rule out obstructive coronary artery disease.

**Figure 2:**
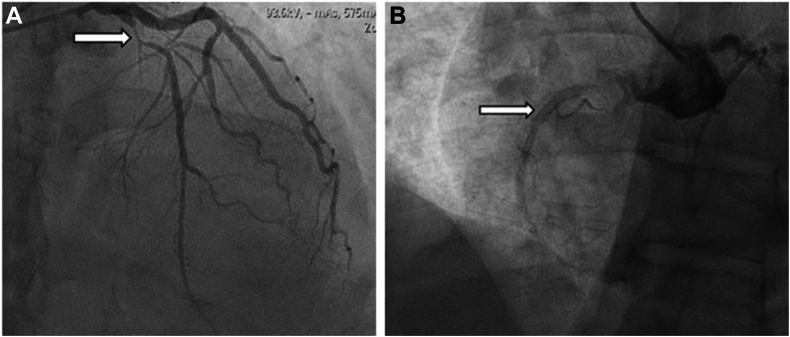
**Left heart catheterizationfindings.** (A) Left heart catheterization (LHC) shows 90% stenosis in the proximal left anterior descending artery, indicated by a white arrow. (B) The anomalous right coronary artery coming from the left cusp seen in LHC is indicated by a white arrow.

**Figure 3:**
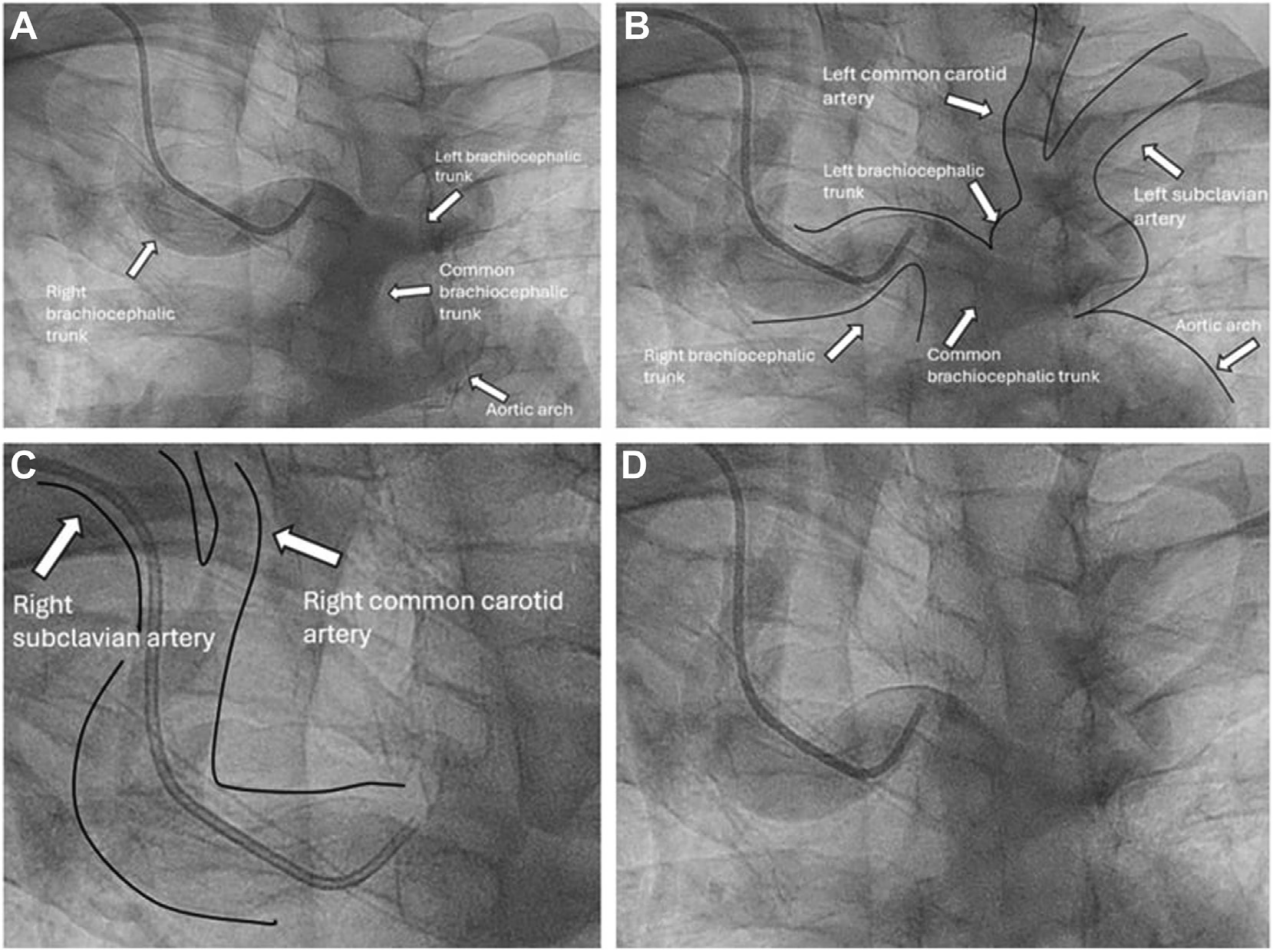
**Unusual bovine arch manifestation.** (A) A common brachiocephalic trunk is seen arising from the aortic arch, splitting into the right and left brachiocephalic trunks. (B) The splitting of the left brachiocephalic trunk into the left common carotid and left subclavian arteries has been traced and drawn out, given the suboptimal contrast injection on left heart catheterization (LHC). (C) The splitting of the right brachiocephalic trunk into the right common carotid and right subclavian arteries has been traced and drawn out, given the suboptimal contrast injection on LHC. (D) The splitting of the left and right brachiocephalic trunks can be faintly discerned in the absence of drawings.

On admission, vital signs were stable. Initial labs were remarkable for a cholesterol of 213 mg/dL, a low-density lipoprotein level of 123 mg/dL, and an HbA1c of 5.7%. Initial electrocardiogram showed sinus bradycardia (heart rate of 55 beats per minute) with a prolonged QT interval of 472 ms. In the angiogram, the LCA was engaged with a contralateral left support guide catheter, whereas the RCA was nonselectively engaged with an Amplatz left guide catheter. The latter was furthermore done given the turbulent flow that was observed during the LHC. The LHC was consistent with 90% stenosis of the proximal LAD (Figure 2A), without any significant stenosis of the left main coronary artery, the LCx artery and its branches, and the RCA, which was seen to have an anterior take-off from the left cusp (Figure 2B). An unusual bovine arch with a common brachiocephalic trunk splitting into left and right brachiocephalic trunks was also observed (Figure 3A-D; Supplemental Video 1). No additional physiologic assessment was done in the LAD or the RCA, as the patient already had a positive stress test (demonstrating anterior ischemia), and the LCx was angiographically normal.

**Figure 4:**
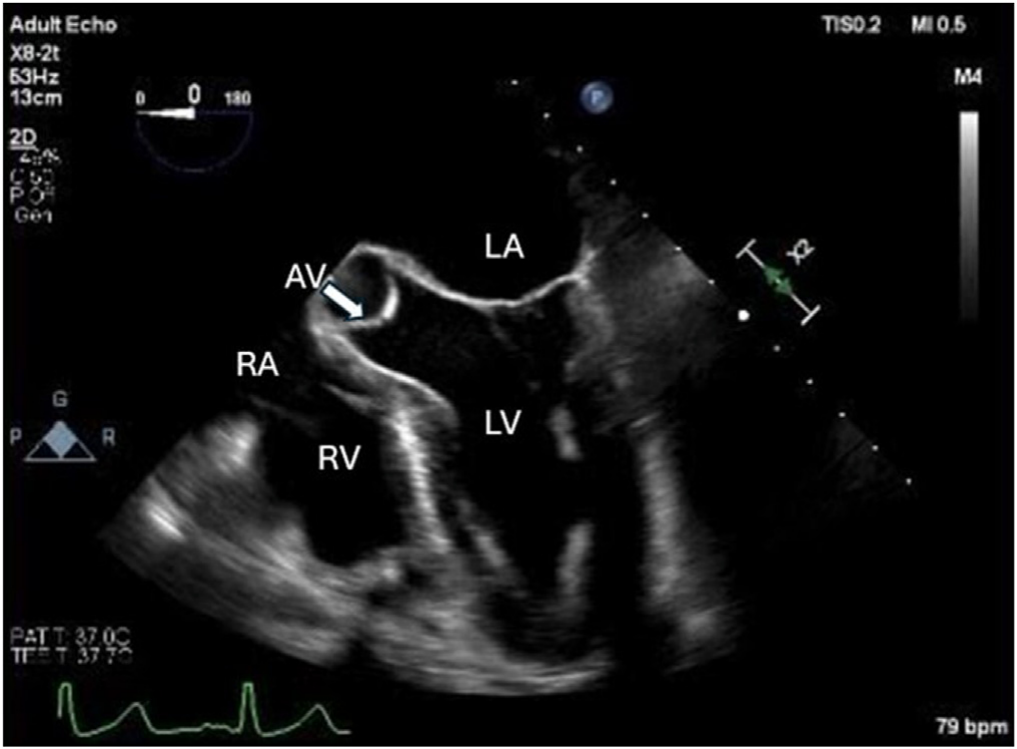
**Transesophageal echocardiogramimage obtained during surgery.** The 4 chambers of the heart are labeled along with the aortic valve. AV, aortic valve; LA, left atrium; LV, left ventricle; RA, right atrium; RV, right ventricle.

Taking into consideration the American Heart Association guidelines for the management of adults with congenital diseases, which recommend surgical intervention in patients with ACAOS who present with ischemic symptoms during diagnostic testing, a shared decision in consultation with cardiothoracic surgery was made.^5^ The teams decided that, given the concerning intramural and interarterial course of this patient’s RCA, there was a need for unroofing. Moreover, due to the patient’s stenotic LAD, a decision was made to proceed with a minimally invasive coronary artery bypass graft (MICABG) over percutaneous coronary intervention as MICABG would further minimize future need for reintervention and given that it is associated with better survival outcomes.^6^ For this reason, the patient underwent a left internal mammary arterial graft to LAD anastomosis. A bypass of the posterior descending artery was not considered, given that there was no identified lesion in the posterior descending artery during the LHC. Pre- and postsurgery transesophageal echocardiogram demonstrated a left ventricular ejection fraction of 55% and 60%, respectively. No thrombi were visualized (Figure 4; Supplemental Videos 2 and 3).

The patient remained hemodynamically stable and was extubated overnight without any issues. He was discharged on postoperative day 3 on amiodarone following 2 episodes of atrial fibrillation with rapid ventricular response heart rate of 160), metoprolol tartrate, along with aspirin, atorvastatin, and clopidogrel.

## Discussion

This case represents the first reported coexistence of an anomalous RCA that begins at the origin of the left cusp and a rare bovine arch variant in a patient without tetralogy of Fallot. The unusual bovine arch with a common brachiocephalic trunk adds to the diversity of AA variants and raises the question of whether this presentation represents a new classification. Although a single aortic angiogram was performed, the observed AA morphology of our patients alludes to the AA variant which is seen in cattle, and not to a fifth persistent AA morphology as demonstrated by the improved persistent fifth aortic arch classification system in the paper by Shan et al.^7^

As previously mentioned, multiple congenital variations of the AA exist, ranging from asymptomatic to symptomatic anomalies. These arch variations include reported cases where the left vertebral artery arises directly from the AA, or cases in which the right subclavian artery originates from the distal arch or proximal descending aorta.^8^ Due to their complex AA anatomy, patients with bovine arches are at an increased risk for technical difficulties and interventional complications. Studies evaluating patients undergoing carotid artery stenting of the left internal carotid artery found that patients with bovine arches had increased catheter manipulation time and adverse cardiovascular and cerebral events compared with patients with nonbovine anatomy.^9^ However, patients with bovine arches undergoing carotid artery stenting of the left internal carotid artery under cerebral protection, using a distal filter or a proximal protection device via a transradial or transbrachial approach, have been reported to have promising clinical success with limited adverse outcomes.^10^

Although bovine arches are considered clinically insignificant, just like in this patient where it was an incidental finding, their association with left-sided strokes, aortic aneurysms, and aortic dissections may necessitate further exploration.^3^ However, it is possible that the concurrent coexistence of a bovine arch and an anomalous RCA may also not present any significant clinical impact in certain patients, other than the individualized risk component of ACAOS.

The management of ACAOS is guided by a patient’s individual risk of ischemia and SCD. In our case, the malignant intramural and interarterial course of the RCA warranted surgical unroofing, whereas the 90% to 95% proximal LAD stenosis necessitated revascularization via MICABG. The idea of the possible need for LAD reintervention and the fact that the patient would necessitate unroofing was what convinced the teams toward a one-time surgical procedure. Although our patient may not fit the typical profile of younger individuals or athletes with ACAOS, his symptoms could not be disregarded, given an elevated risk for ischemia.

## Conclusion

This case emphasizes the need for a tailored, multidisciplinary approach, integrating advanced imaging, evidence-based guidelines, and collaborative decision-making to manage the coexistence of anomalous coronary and AA anatomy. By sharing insights from such cases, we hope to contribute to more precise risk stratification, individualized treatment strategies, and improved outcomes in patients with these rare but significant conditions.

## References

[1] Driesen B.W., Warmerdam E.G., Sieswerda G.T. (2018). Anomalous coronary artery originating from the opposite sinus of Valsalva (ACAOS), fractional flow reserve- and intravascular ultrasound-guided management in adult patients. Catheter Cardiovasc Interv.

[2] Blomjous M.S.H., Budde R.P.J., Bekker M.W.A. (2021). Clinical outcome of anomalous coronary artery with interarterial course in adults: single-center experience combined with a systematic review. Int J Cardiol.

[3] Rotundu A., Nedelcu A.H., Tepordei R.T. (2024). Medical-surgical implications of branching variation of human aortic arch known as bovine aortic arch (BAA). J Pers Med.

[4] Kakucs Z., Heidenhoffer E., Pop M. (2022). Detection of coronary artery and aortic arch anomalies in patients with tetralogy of fallot using CT angiography. J Clin Med.

[5] Stout K.K., Daniels C.J., Aboulhosn J.A. (2019). 2018 AHA/ACC Guideline for the management of adults with congenital heart disease: executive summary: a report of the American College of Cardiology/American Heart Association Task Force on Clinical Practice Guidelines. J Am Coll Cardiol.

[6] Habib R.H., Dimitrova K.R., Badour S.A. (2015). CABG versus PCI: Greater Benefit in Long-Term Outcomes With Multiple Arterial Bypass Grafting. J Am Coll Cardiol.

[7] Shan H., Du X., Zheng G., Ke T., Liao C., Yang H. (2023). Persistent fifth aortic arch: a comprehensive literature review. Front Pediatr.

[8] Onrat E., Uluışık I.E., Ortug G. (2021). The left vertebral artery arising directly from the aortic arch. Transl Res Anat.

[9] Burzotta F., Nerla R., Pirozzolo G. (2015). Clinical and procedural impact of aortic arch anatomic variants in carotid stenting procedures. Catheter Cardiovasc Interv.

[10] Montorsi P., Galli S., Ravagnani P.M. (2014). Carotid artery stenting in patients with left ICA stenosis and bovine aortic arch: a single-center experience in 60 consecutive patients treated via the right radial or brachial approach. J Endovasc Ther.

