# Supplementary material for: Concurrent Presentation of an Anomalous Right Coronary Artery and an Unusual Bovine Arch: Case Report
Source: J Soc Cardiovasc Angiogr Interv. 2025 Jul 23;4(8):103759. doi: 10.1016/j.jscai.2025.103759 (PMC12462142; doi:10.1016/j.jscai.2025.103759)
Supplement: Supplementary Material [file mmc1.pptx]

## Slide 1
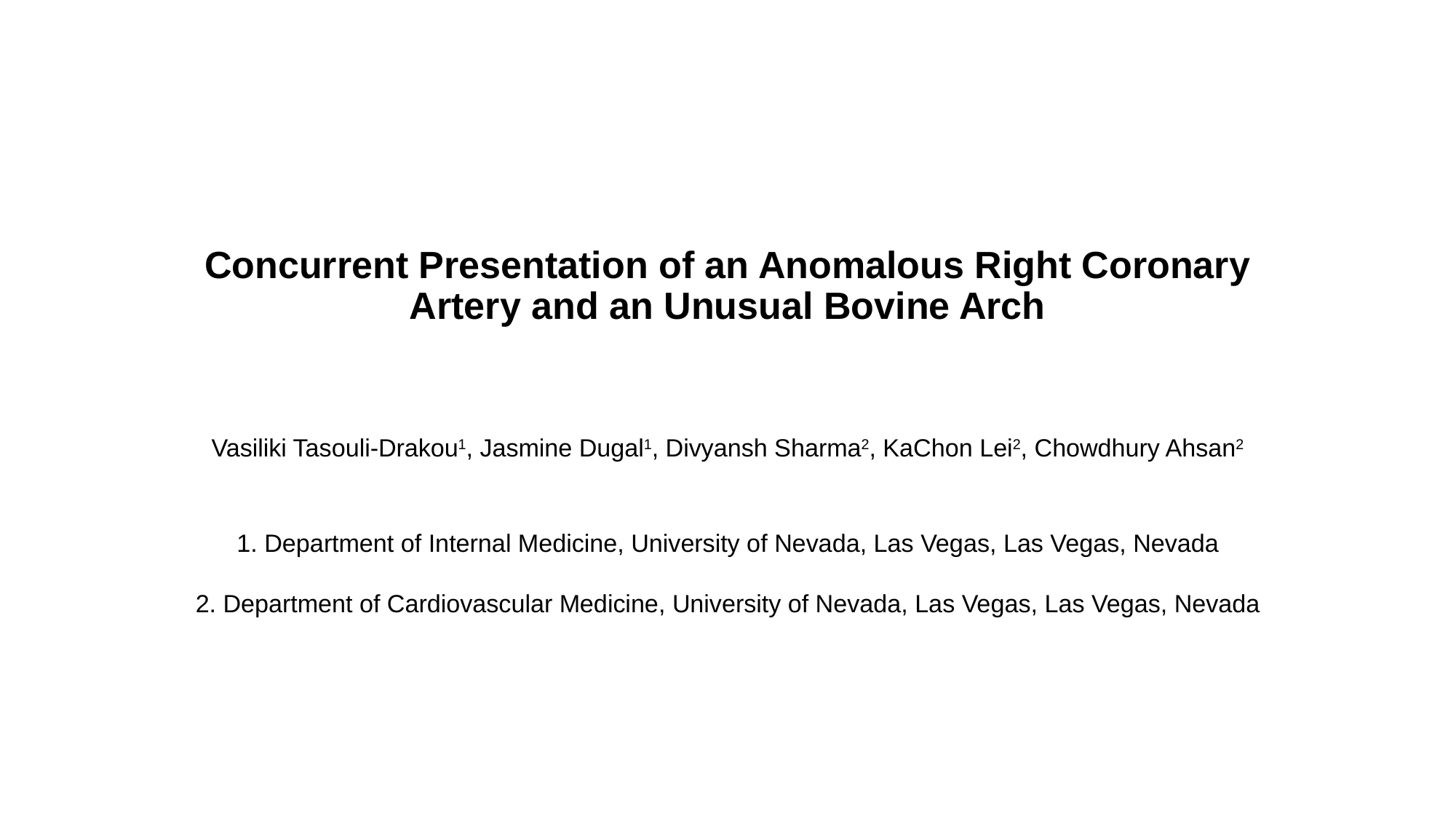

# Concurrent Presentation of an Anomalous Right Coronary Artery and an Unusual Bovine Arch
Vasiliki Tasouli-Drakou1, Jasmine Dugal1, Divyansh Sharma2, KaChon Lei2, Chowdhury Ahsan2
1. Department of Internal Medicine, University of Nevada, Las Vegas, Las Vegas, Nevada
2. Department of Cardiovascular Medicine, University of Nevada, Las Vegas, Las Vegas, Nevada

## Slide 2
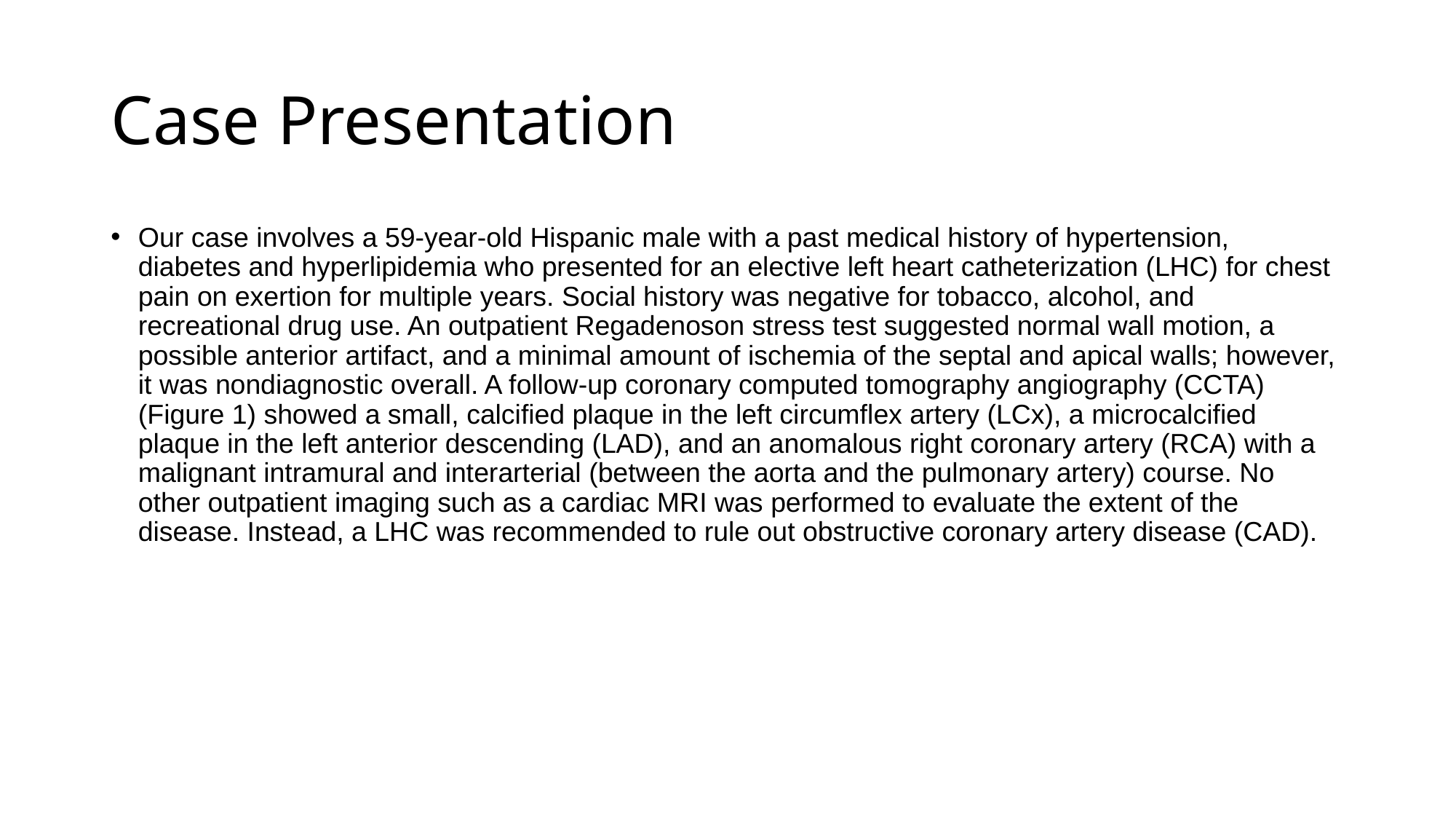

# Case Presentation
Our case involves a 59-year-old Hispanic male with a past medical history of hypertension, diabetes and hyperlipidemia who presented for an elective left heart catheterization (LHC) for chest pain on exertion for multiple years. Social history was negative for tobacco, alcohol, and recreational drug use. An outpatient Regadenoson stress test suggested normal wall motion, a possible anterior artifact, and a minimal amount of ischemia of the septal and apical walls; however, it was nondiagnostic overall. A follow-up coronary computed tomography angiography (CCTA) (Figure 1) showed a small, calcified plaque in the left circumflex artery (LCx), a microcalcified plaque in the left anterior descending (LAD), and an anomalous right coronary artery (RCA) with a malignant intramural and interarterial (between the aorta and the pulmonary artery) course. No other outpatient imaging such as a cardiac MRI was performed to evaluate the extent of the disease. Instead, a LHC was recommended to rule out obstructive coronary artery disease (CAD).

## Slide 3
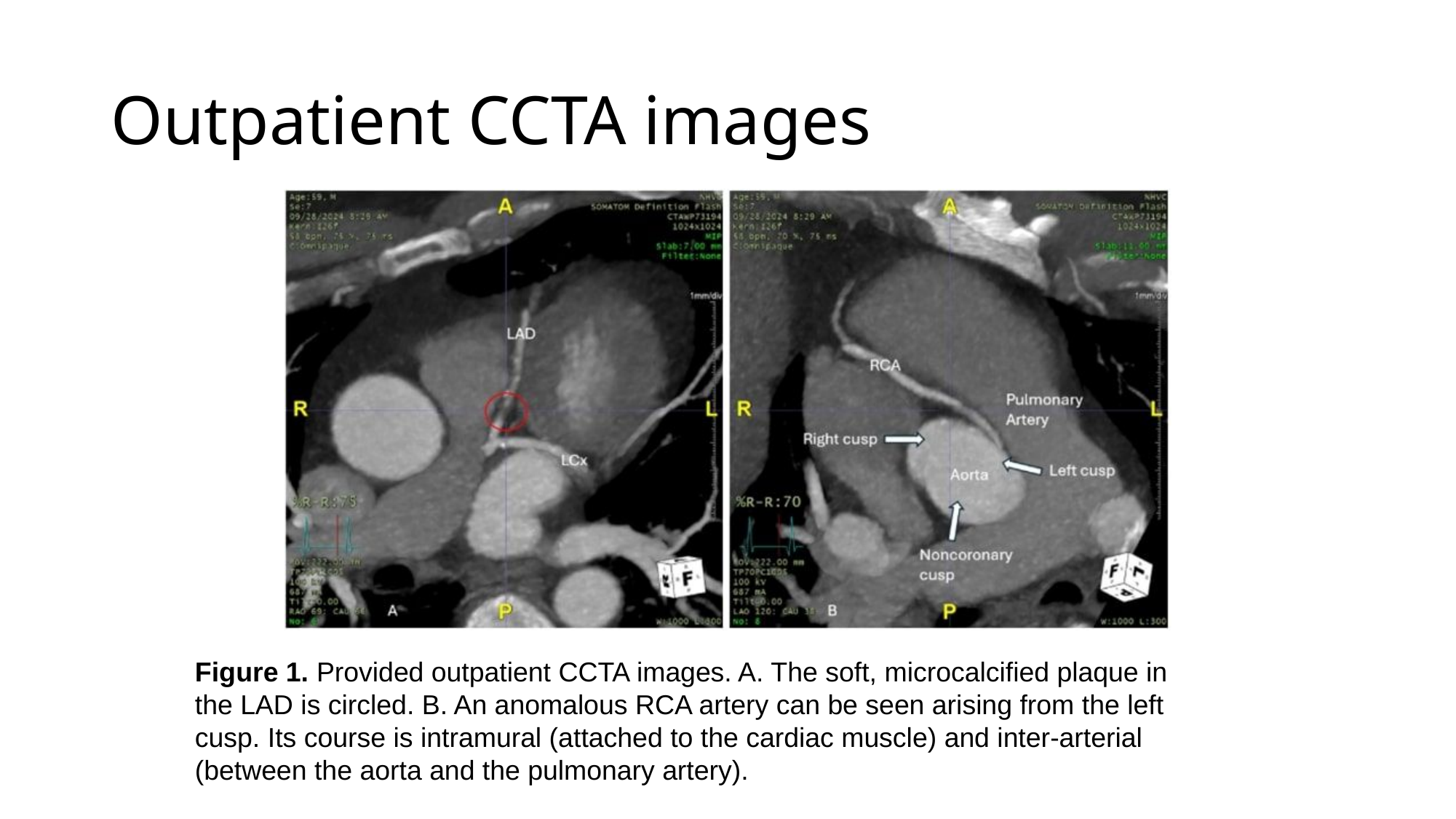

# Outpatient CCTA images
Figure 1. Provided outpatient CCTA images. A. The soft, microcalcified plaque in the LAD is circled. B. An anomalous RCA artery can be seen arising from the left cusp. Its course is intramural (attached to the cardiac muscle) and inter-arterial (between the aorta and the pulmonary artery).

## Slide 4
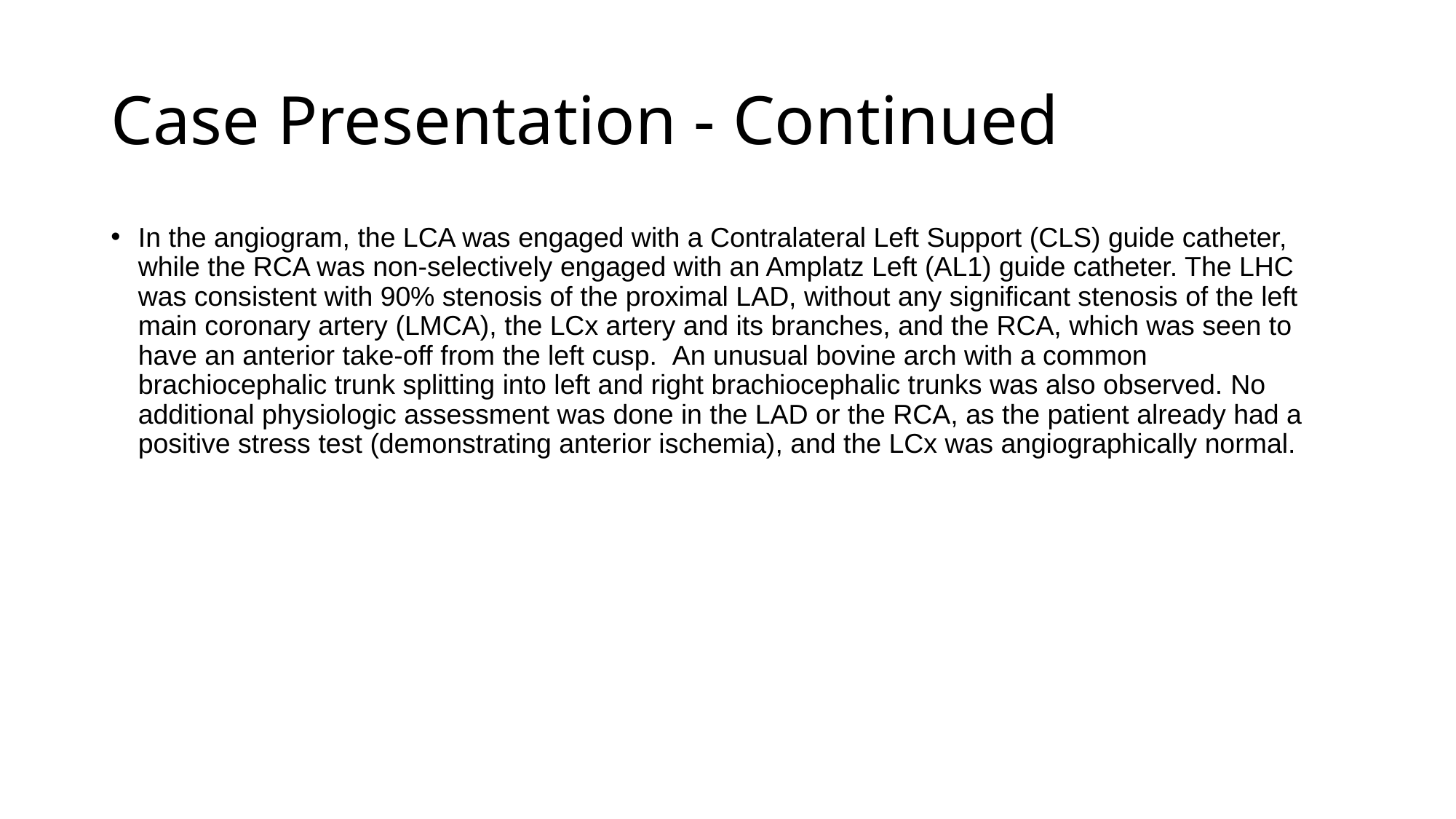

# Case Presentation - Continued
In the angiogram, the LCA was engaged with a Contralateral Left Support (CLS) guide catheter, while the RCA was non-selectively engaged with an Amplatz Left (AL1) guide catheter. The LHC was consistent with 90% stenosis of the proximal LAD, without any significant stenosis of the left main coronary artery (LMCA), the LCx artery and its branches, and the RCA, which was seen to have an anterior take-off from the left cusp.  An unusual bovine arch with a common brachiocephalic trunk splitting into left and right brachiocephalic trunks was also observed. No additional physiologic assessment was done in the LAD or the RCA, as the patient already had a positive stress test (demonstrating anterior ischemia), and the LCx was angiographically normal.

## Slide 5
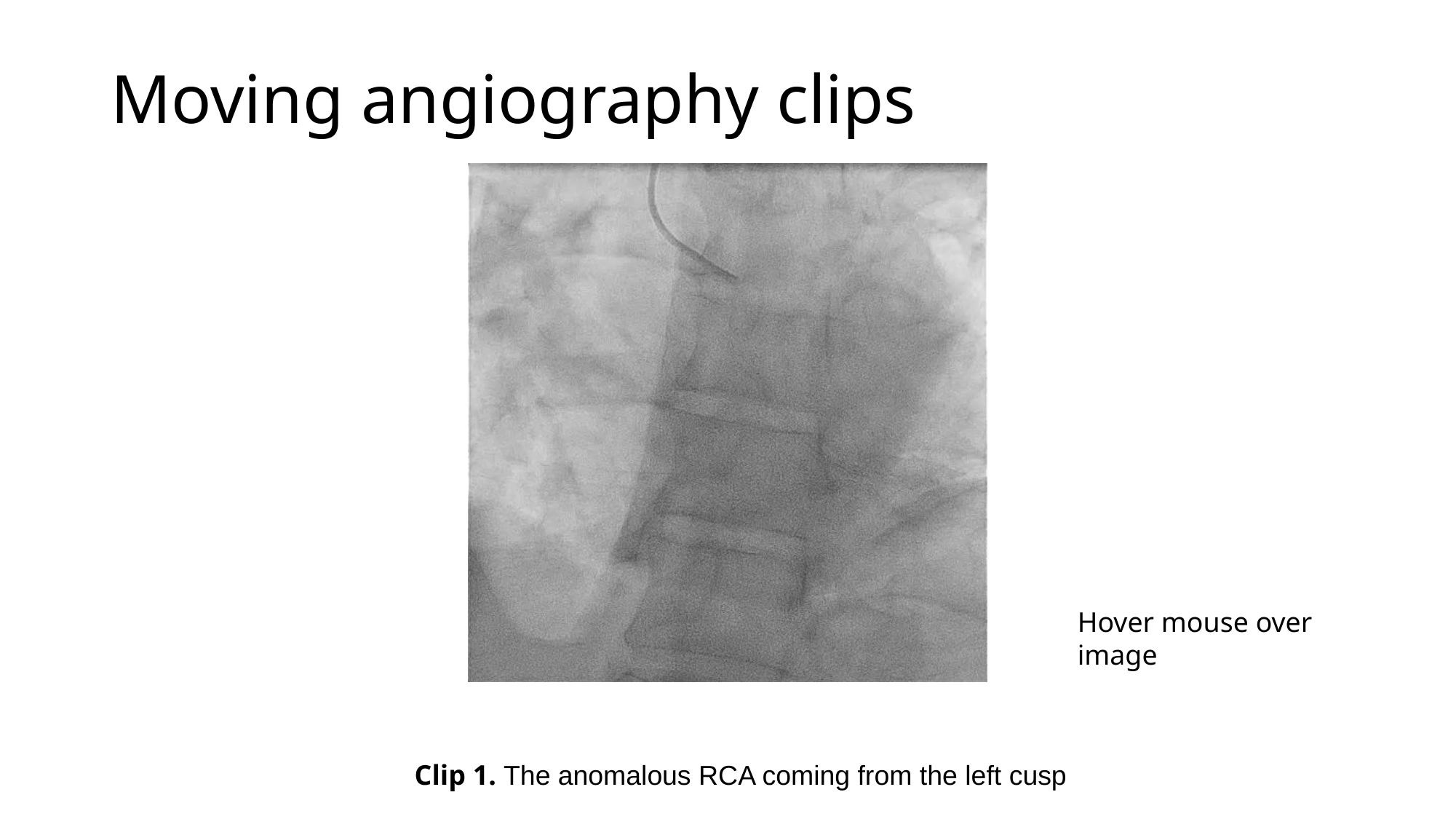

# Moving angiography clips
Hover mouse over image
Clip 1. The anomalous RCA coming from the left cusp

## Slide 6
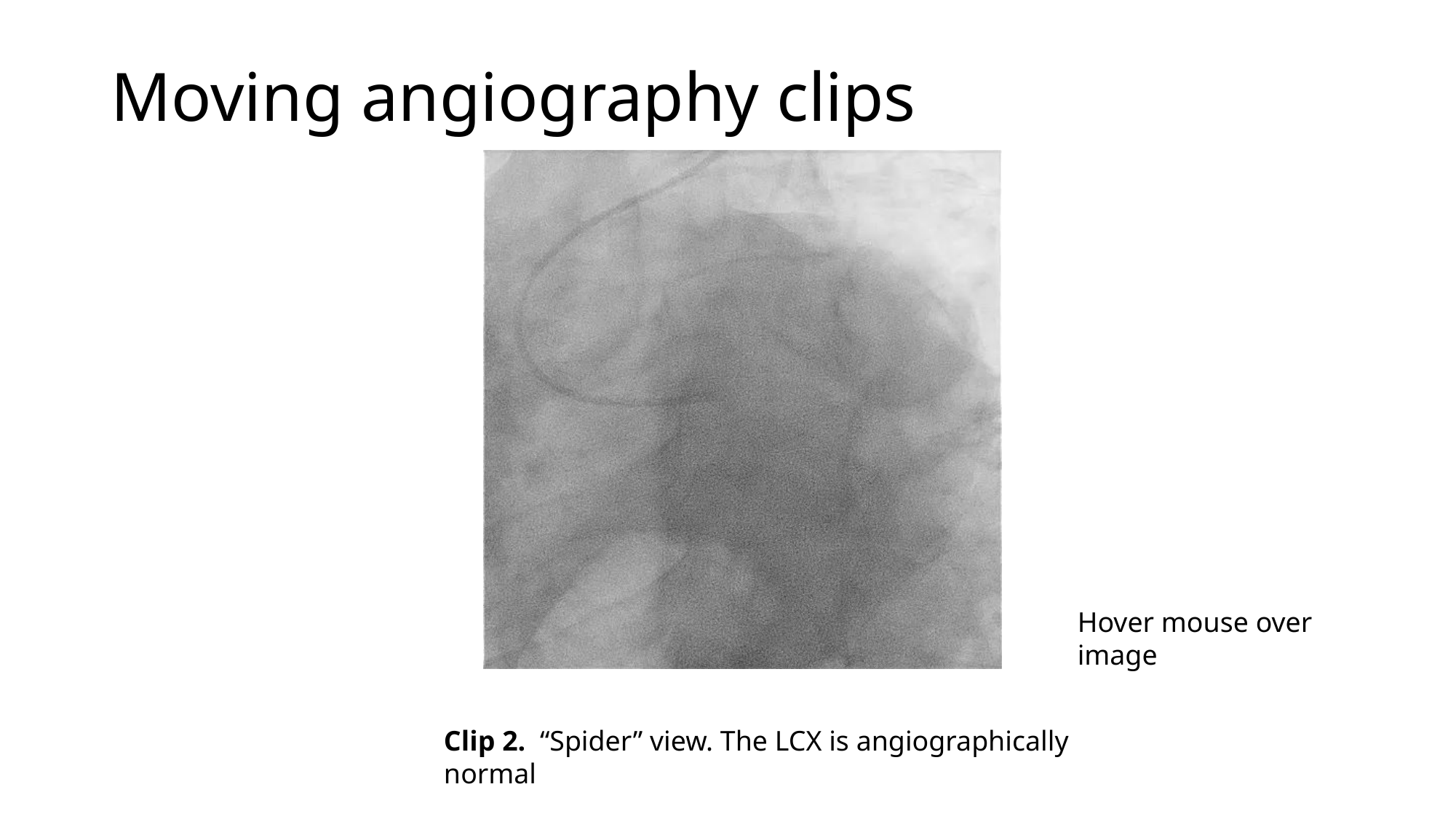

# Moving angiography clips
Hover mouse over image
Clip 2. “Spider” view. The LCX is angiographically normal

## Slide 7
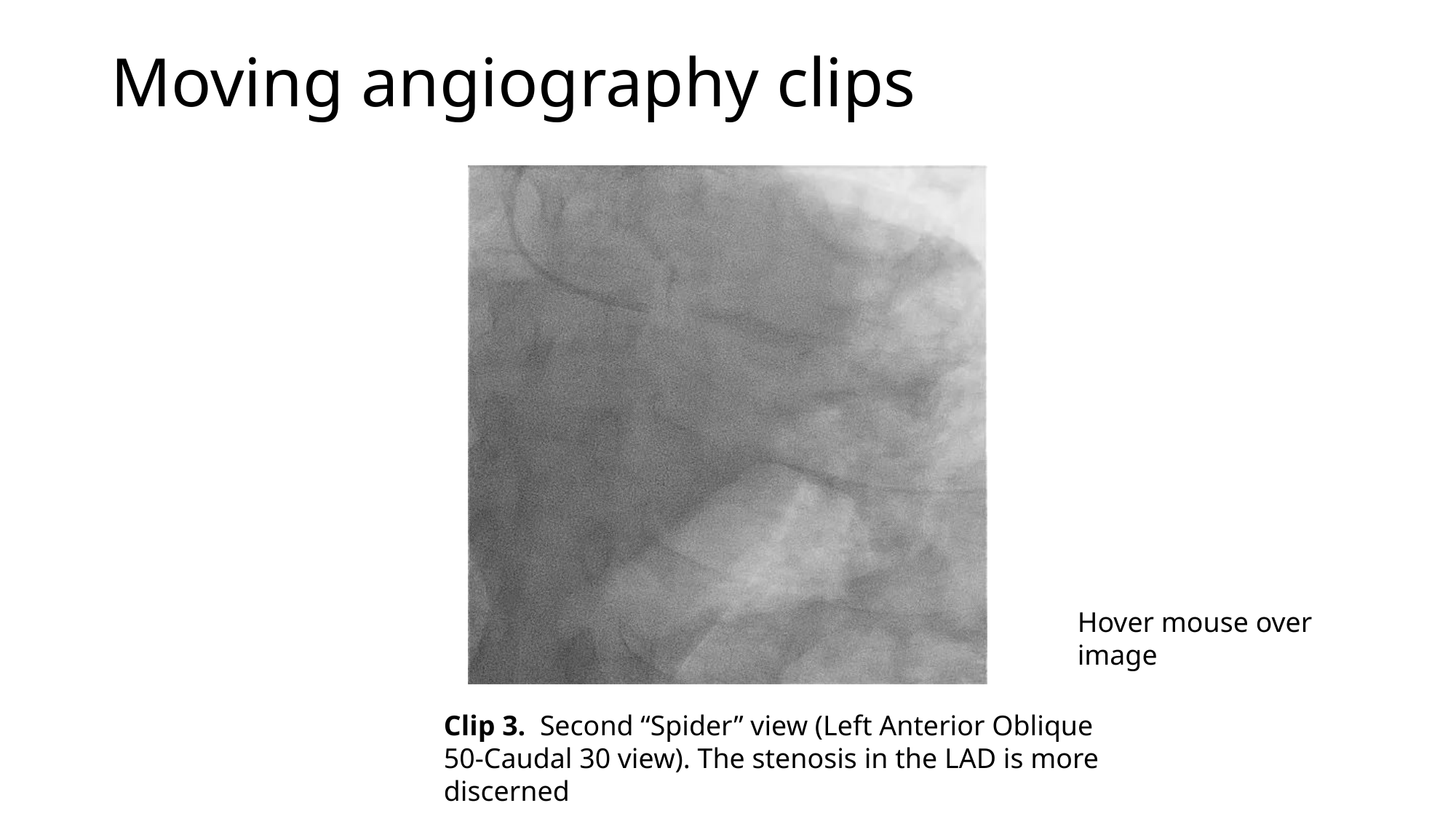

# Moving angiography clips
Hover mouse over image
Clip 3. Second “Spider” view (Left Anterior Oblique 50-Caudal 30 view). The stenosis in the LAD is more discerned

## Slide 8
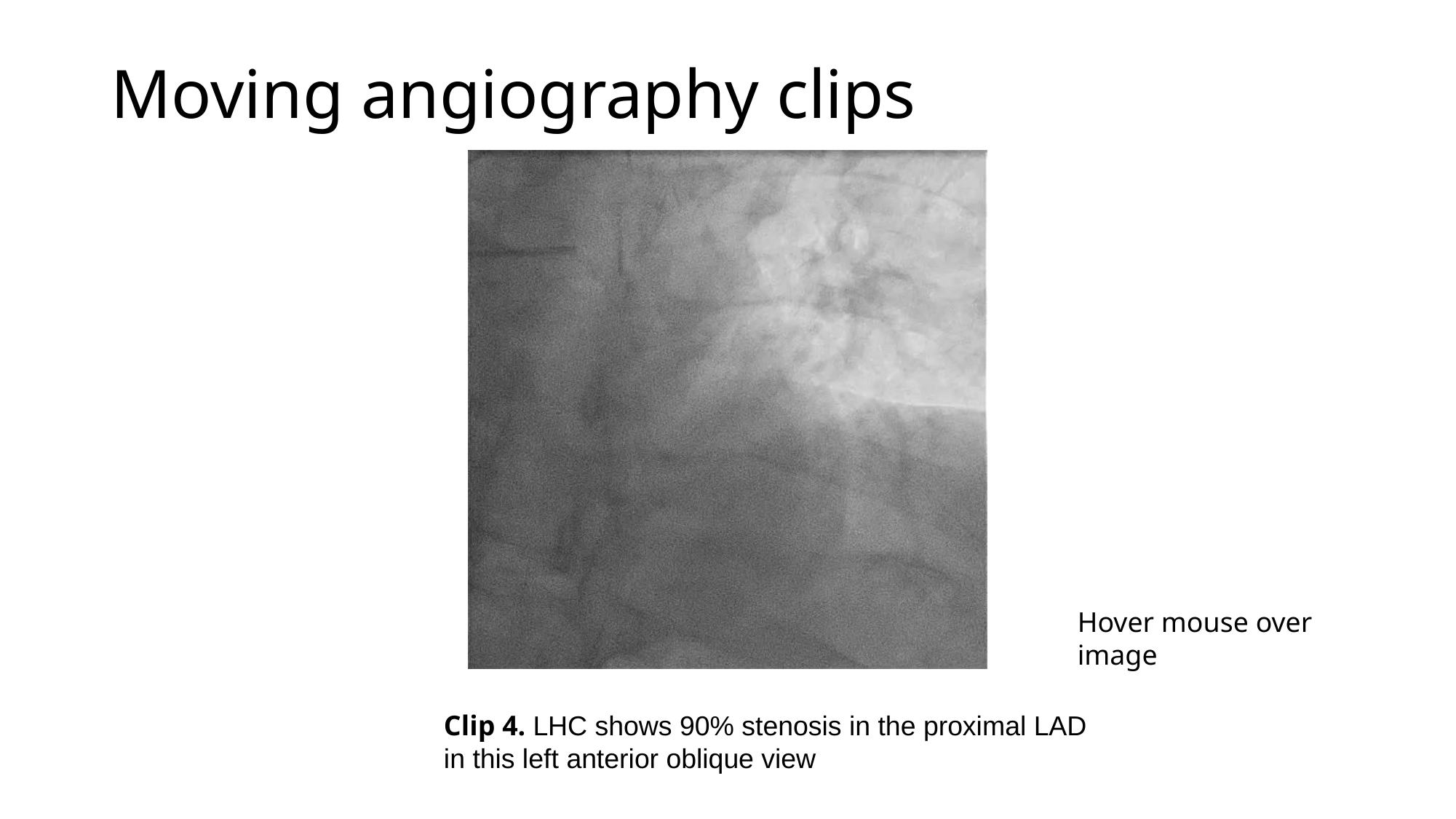

# Moving angiography clips
Hover mouse over image
Clip 4. LHC shows 90% stenosis in the proximal LAD in this left anterior oblique view

## Slide 9
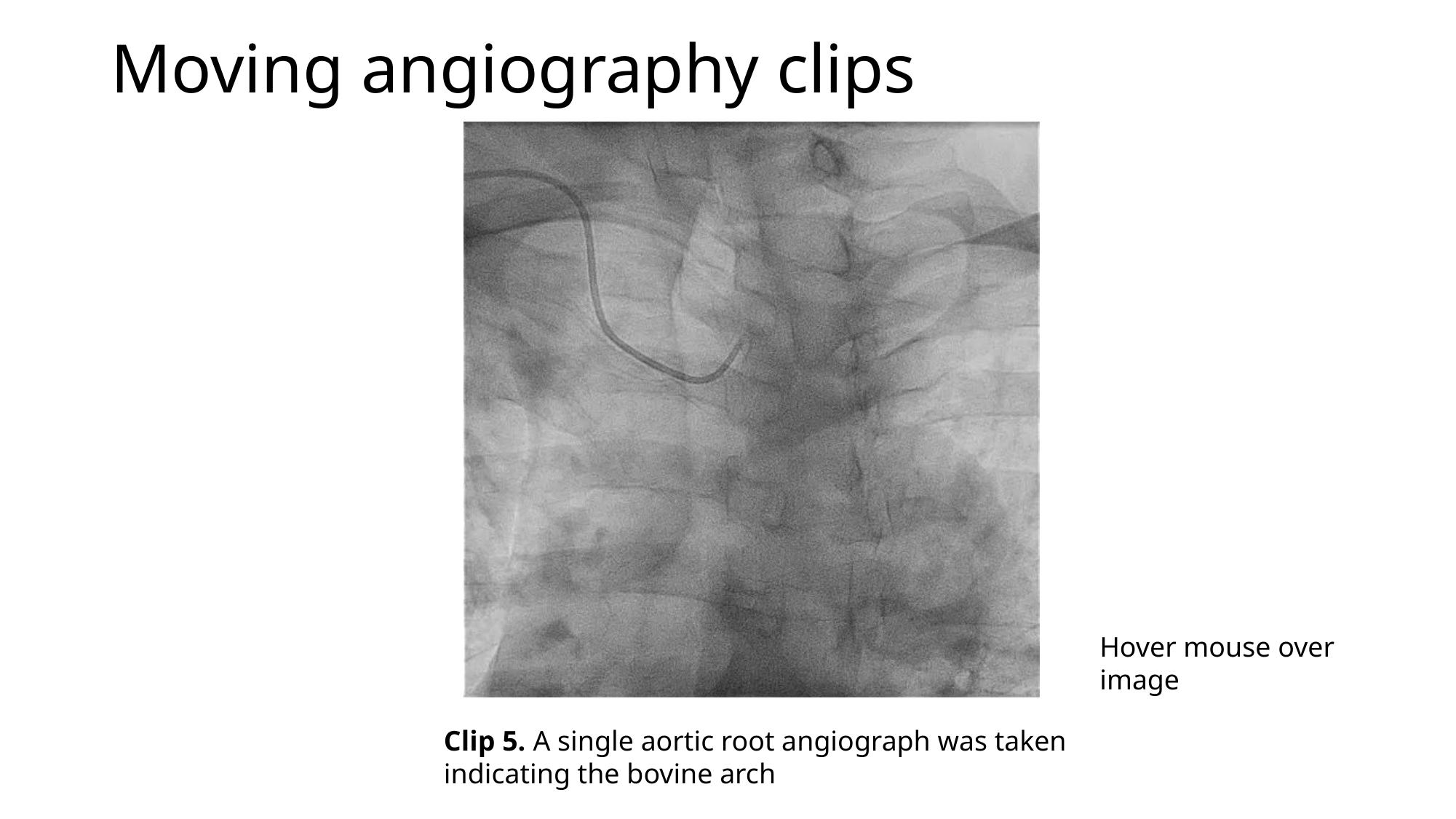

# Moving angiography clips
Hover mouse over image
Clip 5. A single aortic root angiograph was taken indicating the bovine arch

## Slide 10
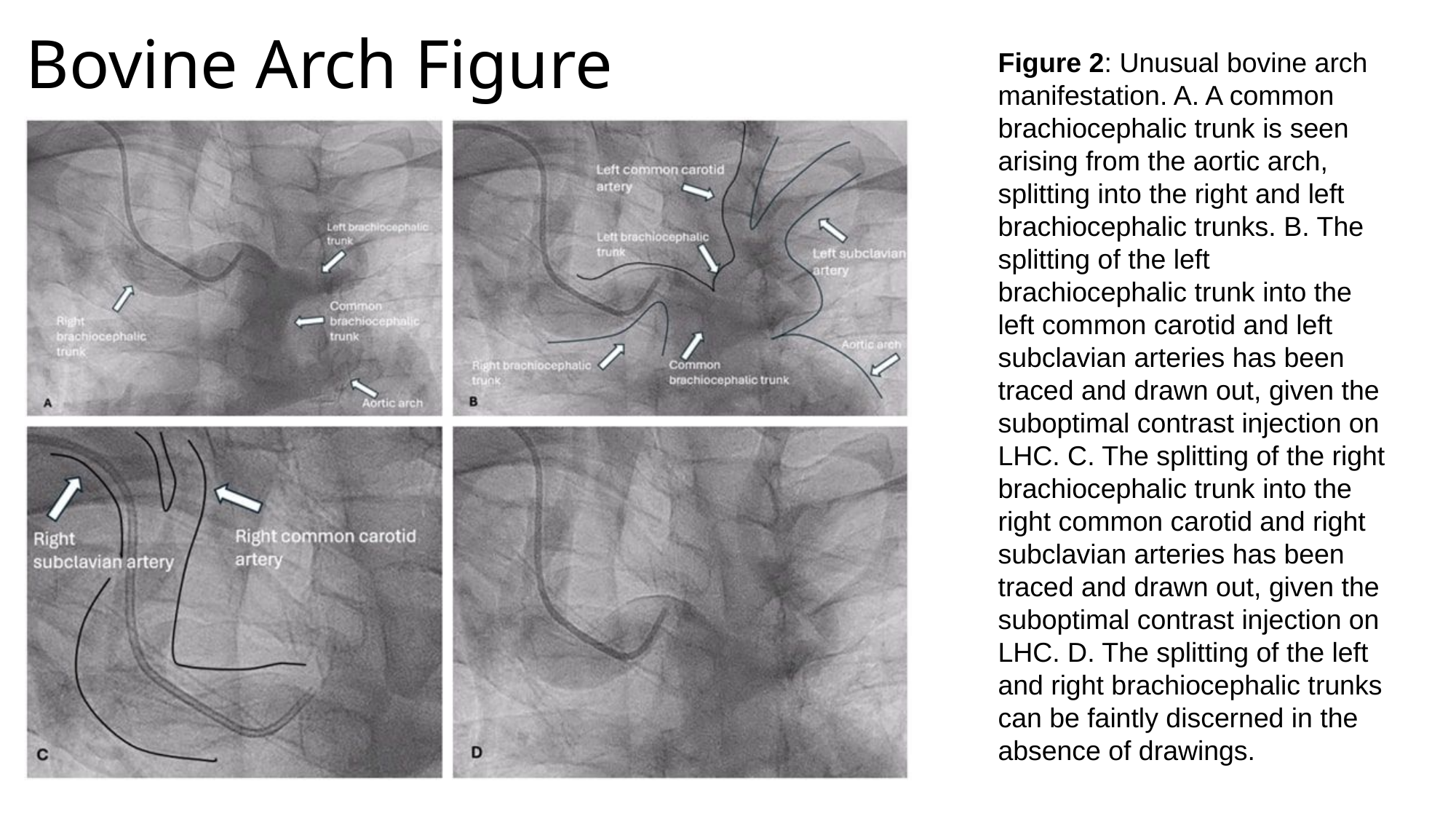

# Bovine Arch Figure
Figure 2: Unusual bovine arch manifestation. A. A common brachiocephalic trunk is seen arising from the aortic arch, splitting into the right and left brachiocephalic trunks. B. The splitting of the left brachiocephalic trunk into the left common carotid and left subclavian arteries has been traced and drawn out, given the suboptimal contrast injection on LHC. C. The splitting of the right brachiocephalic trunk into the right common carotid and right subclavian arteries has been traced and drawn out, given the suboptimal contrast injection on LHC. D. The splitting of the left and right brachiocephalic trunks can be faintly discerned in the absence of drawings.

## Slide 11
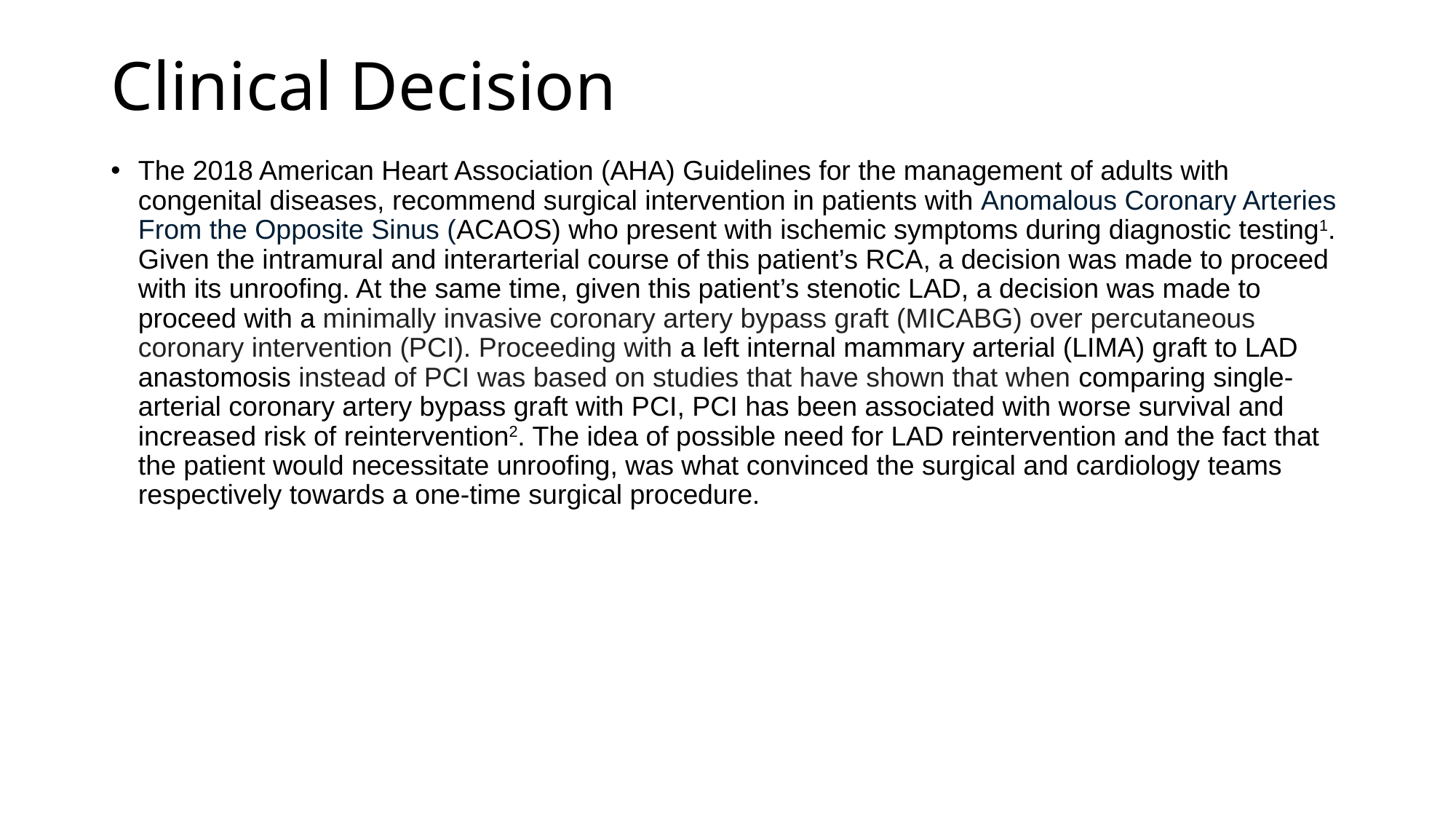

# Clinical Decision
The 2018 American Heart Association (AHA) Guidelines for the management of adults with congenital diseases, recommend surgical intervention in patients with Anomalous Coronary Arteries From the Opposite Sinus (ACAOS) who present with ischemic symptoms during diagnostic testing1. Given the intramural and interarterial course of this patient’s RCA, a decision was made to proceed with its unroofing. At the same time, given this patient’s stenotic LAD, a decision was made to proceed with a minimally invasive coronary artery bypass graft (MICABG) over percutaneous coronary intervention (PCI). Proceeding with a left internal mammary arterial (LIMA) graft to LAD anastomosis instead of PCI was based on studies that have shown that when comparing single-arterial coronary artery bypass graft with PCI, PCI has been associated with worse survival and increased risk of reintervention2. The idea of possible need for LAD reintervention and the fact that the patient would necessitate unroofing, was what convinced the surgical and cardiology teams respectively towards a one-time surgical procedure.

## Slide 12
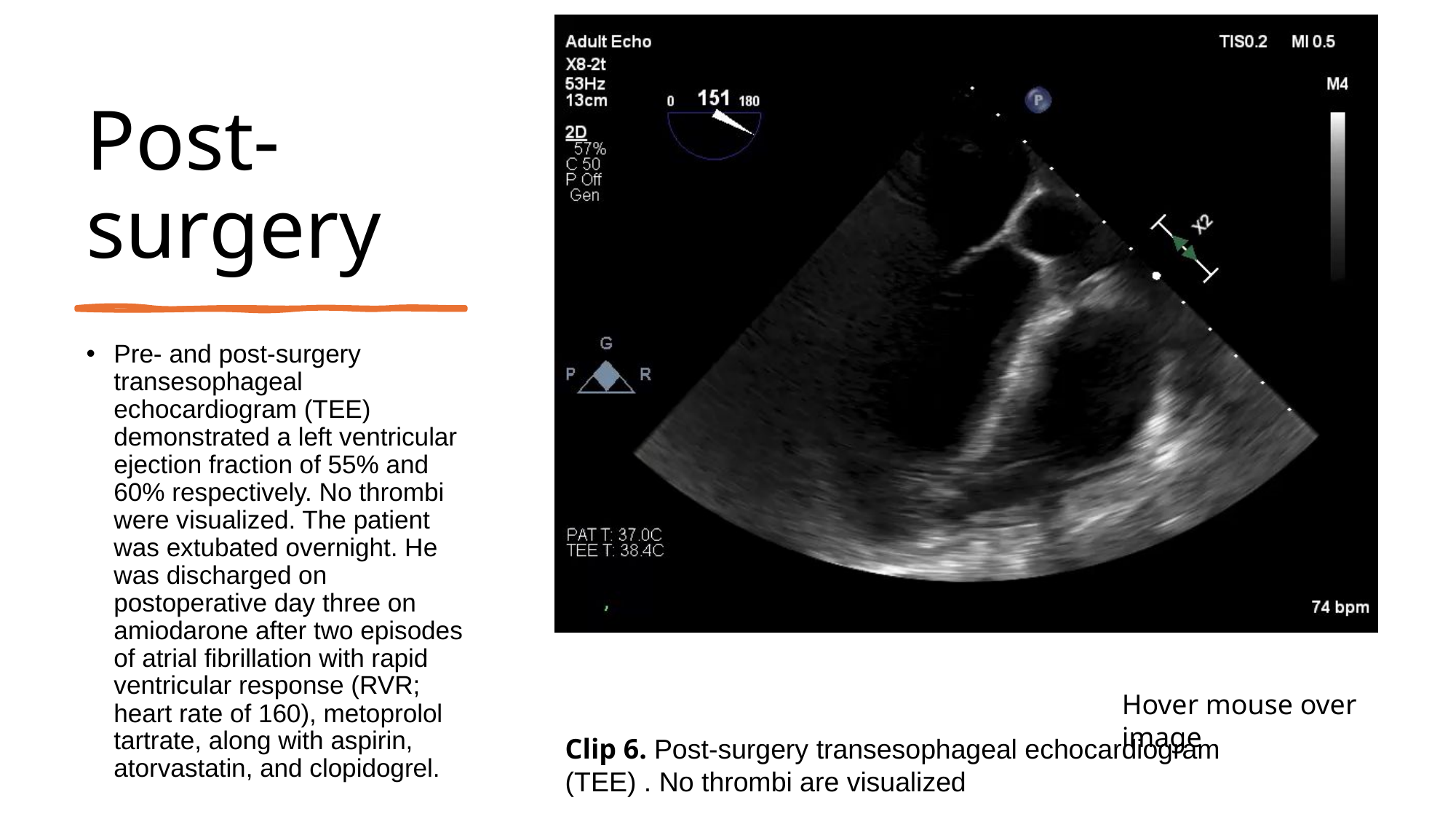

# Post-surgery
Pre- and post-surgery transesophageal echocardiogram (TEE) demonstrated a left ventricular ejection fraction of 55% and 60% respectively. No thrombi were visualized. The patient was extubated overnight. He was discharged on postoperative day three on amiodarone after two episodes of atrial fibrillation with rapid ventricular response (RVR; heart rate of 160), metoprolol tartrate, along with aspirin, atorvastatin, and clopidogrel.
Hover mouse over image
Clip 6. Post-surgery transesophageal echocardiogram (TEE) . No thrombi are visualized

## Slide 13
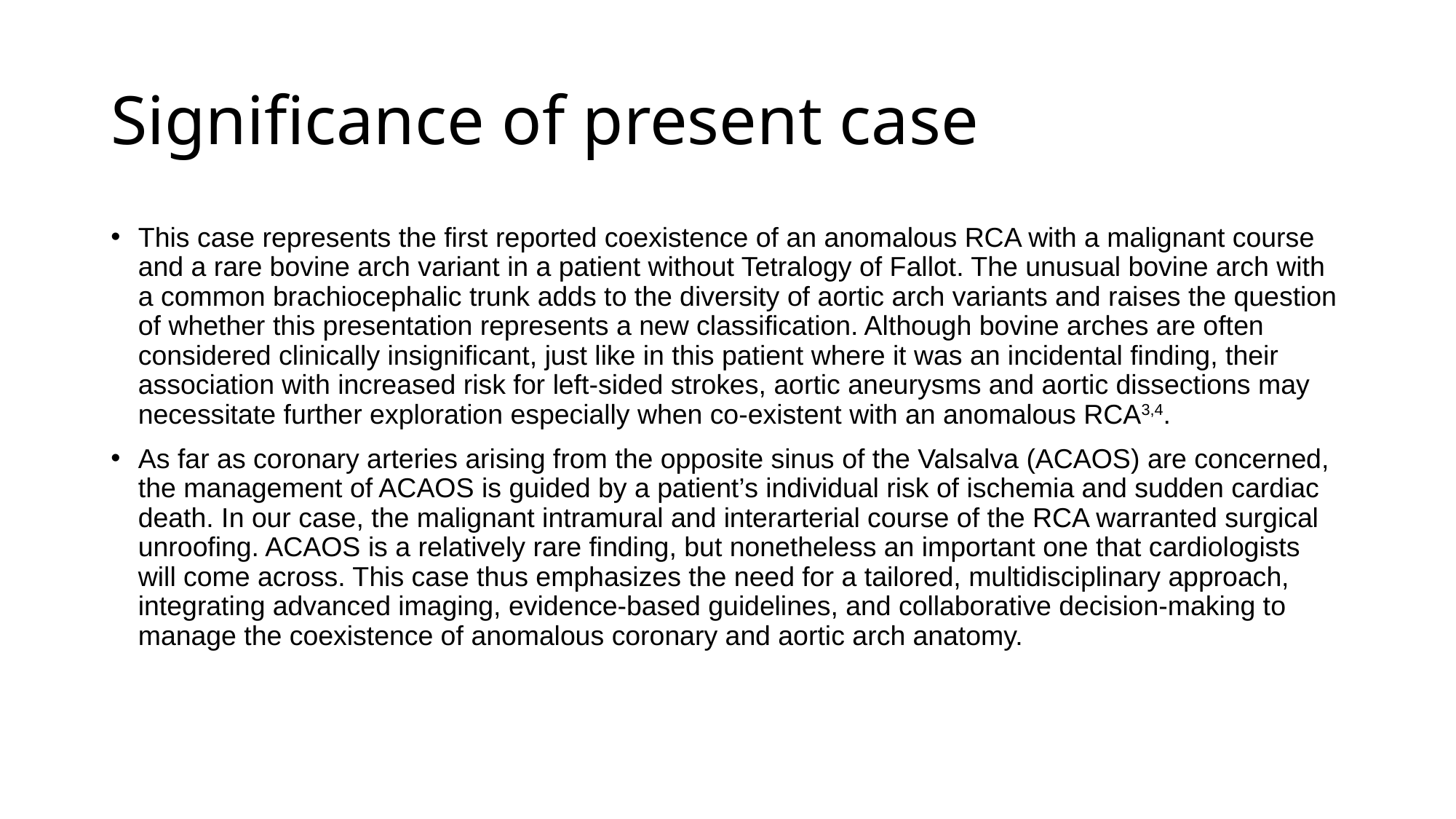

# Significance of present case
This case represents the first reported coexistence of an anomalous RCA with a malignant course and a rare bovine arch variant in a patient without Tetralogy of Fallot. The unusual bovine arch with a common brachiocephalic trunk adds to the diversity of aortic arch variants and raises the question of whether this presentation represents a new classification. Although bovine arches are often considered clinically insignificant, just like in this patient where it was an incidental finding, their association with increased risk for left-sided strokes, aortic aneurysms and aortic dissections may necessitate further exploration especially when co-existent with an anomalous RCA3,4.
As far as coronary arteries arising from the opposite sinus of the Valsalva (ACAOS) are concerned, the management of ACAOS is guided by a patient’s individual risk of ischemia and sudden cardiac death. In our case, the malignant intramural and interarterial course of the RCA warranted surgical unroofing. ACAOS is a relatively rare finding, but nonetheless an important one that cardiologists will come across. This case thus emphasizes the need for a tailored, multidisciplinary approach, integrating advanced imaging, evidence-based guidelines, and collaborative decision-making to manage the coexistence of anomalous coronary and aortic arch anatomy.

## Slide 14
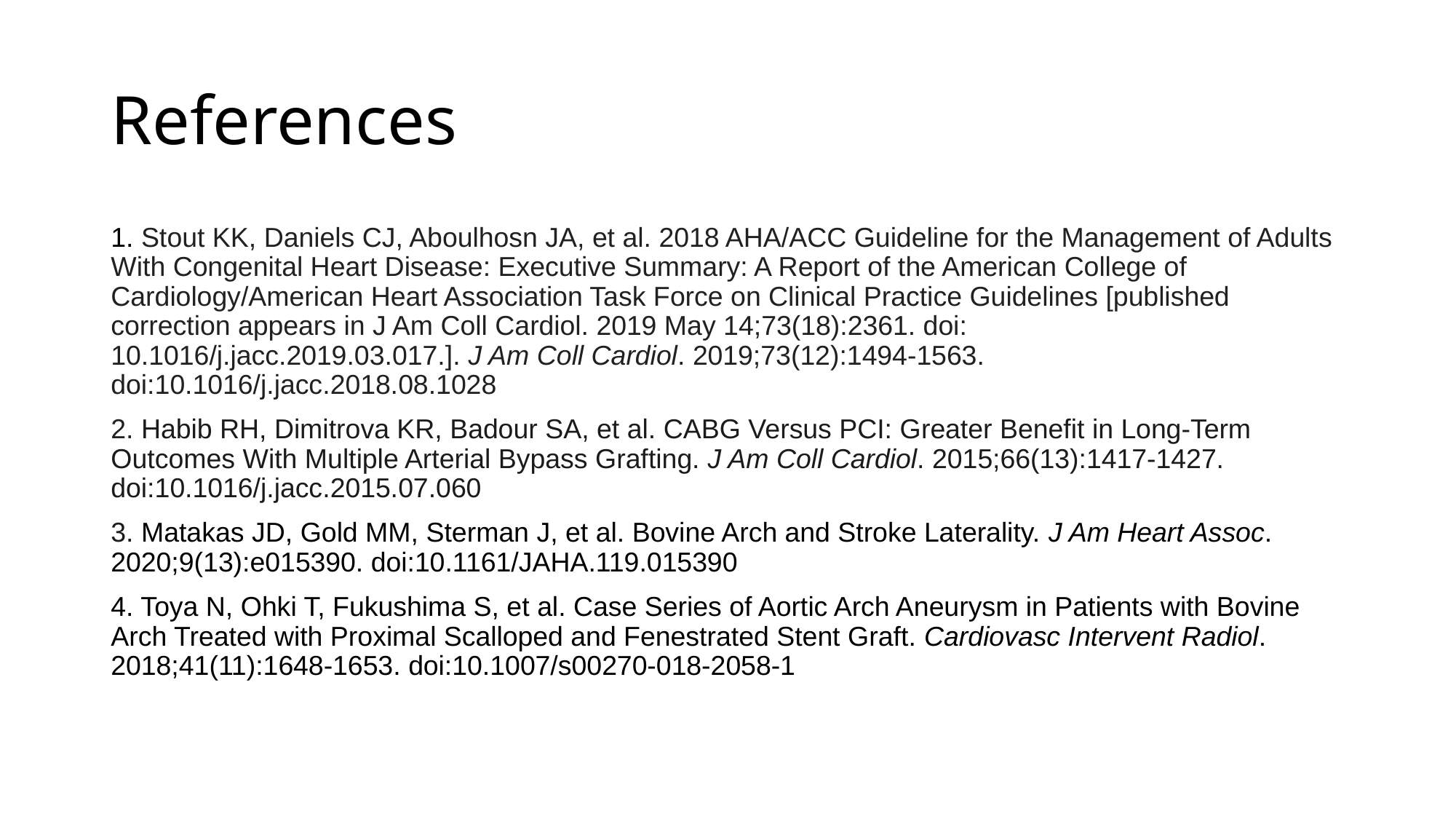

# References
1. Stout KK, Daniels CJ, Aboulhosn JA, et al. 2018 AHA/ACC Guideline for the Management of Adults With Congenital Heart Disease: Executive Summary: A Report of the American College of Cardiology/American Heart Association Task Force on Clinical Practice Guidelines [published correction appears in J Am Coll Cardiol. 2019 May 14;73(18):2361. doi: 10.1016/j.jacc.2019.03.017.]. J Am Coll Cardiol. 2019;73(12):1494-1563. doi:10.1016/j.jacc.2018.08.1028
2. Habib RH, Dimitrova KR, Badour SA, et al. CABG Versus PCI: Greater Benefit in Long-Term Outcomes With Multiple Arterial Bypass Grafting. J Am Coll Cardiol. 2015;66(13):1417-1427. doi:10.1016/j.jacc.2015.07.060
3. Matakas JD, Gold MM, Sterman J, et al. Bovine Arch and Stroke Laterality. J Am Heart Assoc. 2020;9(13):e015390. doi:10.1161/JAHA.119.015390
4. Toya N, Ohki T, Fukushima S, et al. Case Series of Aortic Arch Aneurysm in Patients with Bovine Arch Treated with Proximal Scalloped and Fenestrated Stent Graft. Cardiovasc Intervent Radiol. 2018;41(11):1648-1653. doi:10.1007/s00270-018-2058-1
